# Assessing the perceived effect of non-pharmaceutical interventions on SARS-Cov-2 transmission risk: an experimental study in Europe

**DOI:** 10.1038/s41598-024-55447-1

**Published:** 2024-02-28

**Authors:** Giuseppe Alessandro Veltri, Janina Isabel Steinert, Henrike Sternberg, Matteo M. Galizzi, Barbara Fasolo, Ploutarchos Kourtidis, Tim Büthe, George Gaskell

**Affiliations:** 1 Department of Sociology and Social Research, University of Trento, Via Verdi 26, 38122 Trento, Italy; 2 TUM School of Social Sciences and Technology & TUM School of Medicine and Health, Technical University of Munich, Munich, Germany; 3 TUM School of Social Sciences and Technology & TUM School of Management, Technical University of Munich, Munich, Germany; 4 Department of Psychological and Behavioural Science and LSE Behavioural Lab, London School of Economics and Political Science, London, UK; 5 Department of Management, London School of Economics and Political Science, London, UK; 6 Sanford School of Public Policy, Duke University, Durham, USA; 7 Department of Methodology, London School of Economics and Political Science, London, UK; 8 Munich School of Politics and Public Policy & TUM School of Social Sciences and Technology & TUM School of Management, Technical University of Munich, Munich, Germany

**Keywords:** COVID-19, NPI, DCE, Risk behaviour, Human behaviour, Health policy

## Abstract

We conduct a large (N = 6567) online experiment to measure the features of non-pharmaceutical interventions (NPIs) that citizens of six European countries perceive to lower the risk of transmission of SARS-Cov-2 the most. We collected data in Bulgaria (*n* = 1069), France (*n* = 1108), Poland (*n* = 1104), Italy (*n* = 1087), Spain (*n* = 1102) and Sweden (*n* = 1097). Based on the features of the most widely adopted public health guidelines to reduce SARS-Cov-2 transmission (mask wearing vs not, outdoor vs indoor contact, short vs 90 min meetings, few vs many people present, and physical distancing of 1 or 2 m), we conducted a discrete choice experiment (DCE) to estimate the public’s perceived risk of SARS-CoV-2 transmission in scenarios that presented mutually exclusive constellations of these features. Our findings indicate that participants’ perception of transmission risk was most influenced by the NPI attributes of mask-wearing and outdoor meetings and the least by NPI attributes that focus on physical distancing, meeting duration, and meeting size. Differentiating by country, gender, age, cognitive style (reflective or intuitive), and perceived freight of COVID-19 moreover allowed us to identify important differences between subgroups. Our findings highlight the importance of improving health policy communication and citizens’ health literacy about the design of NPIs and the transmission risk of SARS-Cov-2 and potentially future viruses.

## Introduction

The COVID-19 pandemic, marked by nearly eight hundred million confirmed cases and seven million deaths globally as of November 2023, stands as one of the most significant health crises in recent history^1^. Initially governments around the world relied heavily on non-pharmaceutical interventions (NPIs), that is, public health interventions^2^ such as lockdown, isolation, wearing masks, and practicing physical distancing, as immediate strategies to curb the spread of the virus. The landscape changed with the development of COVID-19 vaccines in late 2020, substantially reducing the risk of severe infection^3^, shifted much of the attention to the vaccines. Yet, even after the advent of COVID-19 vaccines, NPIs continue to be useful tools for public health behaviours.

One of the reasons for the persistence of NPIs in mitigating COVID-19 is the emergence of highly transmissible variants. While epidemiological models initially suggested that herd immunity could be achieved with a 66% vaccination rate, assuming a 90% vaccine efficacy^4^, the Delta variant of SARS-CoV-2 proved twice as transmissible as its predecessors^5^. The increased transmissibility of Delta elevated the herd immunity threshold beyond 90%^6^. With universal vaccine access lacking, and a significant portion of the global population below the vaccination threshold^6,7^, implementation of NPIs, alongside vaccination efforts, remained essential to curb the spread of the virus. The U.S. Center for Disease Control and Prevention (CDC) emphasised this need when it updated its guidance in the summer of 2021, recommending that fully vaccinated individuals wear masks in public indoor spaces^8^. At the same time, research findings further indicated reduced effectiveness of COVID-19 vaccines against the Delta variant compared to earlier strains^9^, highlighting the need for continuous engagement in protective behaviours. Countries that maintained NPIs throughout the pandemic demonstrated more effective control of the Delta outbreak^6^. Taken together, the evidence suggests that NPIs continued to play a crucial role in mitigating COVID-19, even after vaccine rollouts^10^.

Apart from the emergence of variants and herd immunity, the declining immunity against COVID-19 over time^10,11^ and asymptomatic infection, especially among vaccinated populations in the case of Omicron variant^12^ provided additional reasons for the continued implementation of NPIs as did vaccine hesitancy. There is a large body of literature investigating which parts and which characteristics of the public are vaccine-hesitant^13–16^. For these parts of the population, NPIs might be a more suitable means for protecting public health.

Systematic evidence highlights the importance of different NPIs against COVID-19 and previous epidemics^17,18^, even beyond the immediate response to a disease outbreak. Notably, during the COVID-19 pandemic, measures such as mask-wearing, ventilation, avoidance of large gatherings, schools and restaurants closures, and physical distancing emerged as highly effective during the COVID-19 pandemic^10,19–22^. In contrast, NPI measures like contact tracing and isolation appeared to be less effective^23^. Compliance with pandemic rules, however, is not always straightforward, as it can be influenced by sociodemographic factors, behavioural factors, and human perceptions^13,24,25^. In particular, it can be affected by the high heterogeneity in risk perceptions and preferences during public health emergencies^26^. Part of the variability in risk perception can be attributed to factors like age, with older individuals exhibiting higher risk perception and greater adherence to public health rules^26–30^. Gender also plays a role, as females demonstrate higher compliance with COVID-19 guidance^27–31^. In terms of levels of education, higher education is positively associated with both COVID-19 risk perception and adherence to pandemic rules^28–30^. Exposure to crises^32,33^, affective responses to threats^34–36^, and the variety of specific methods used to measure risk perception^37^ further contribute to this heterogeneity.

Recent research at the intersection of risk perception and compliance with pandemic rules highlights a consistent finding; that is, risk perception substantially predicts compliance behaviour^27–30,38^. This aligns with existing health behaviour theories, such as the Health Belief Model^39^ and the Protection Motivation Theory^40^, which posit that health behaviours depend on people’s perceived risk, encompassing the perceived severity of and vulnerability to a health threat. Within the context of protective behaviours during crises, this suggests that higher perceived risk motivates individuals to comply with public health guidelines and associated NPIs^28,41^. Likewise, recent research suggests that a reduction in risk perception that naturally occurred during the COVID-19 pandemic corresponds with a decrease in compliance with public health measures, highlighting the interplay between risk perception and behaviour^38^.

In the evolving landscape of research on risk perception during the COVID-19 pandemic, a critical gap persists regarding the effects of NPIs on individuals’ risk perception concerning SARS-CoV-2 virus transmission. Our research aims to address this gap by conducting a comprehensive assessment of how the most widely adopted NPIs during the COVID-19 pandemic affect people’s risk perception of virus transmission. Concretely, we employ a discrete choice experiment (DCE)^42^, in which the defining characteristics of each NPI becomes an attribute, varied systematically across the choice sets to investigate how participants' risk perception vary in response to specific NPIs. The NPIs included: (a) personal protective equipment—i.e., mask wearing vs. not; (b) settings of a meeting—i.e., indoors vs. outdoors; (c) size of gathering—i.e., two people vs. ten people; (d) duration of contact—i.e., 15 min vs. 90 min; and (e) physical distance—i.e., 1 m vs. 2 m. A detailed discussion of our methods and findings is presented in the subsequent sections.

## Findings

In Supplementary Annex Sects. 1 and 2, we report the full set of descriptive statistics for our sample, including socio-demographic variables, Cognitive Reflective Test (CRT) scores and Perceived Freight of COVID-19 (PFC) scores. None of the distributions shows extreme skewness. We also report the parameter estimates based on a conditional logistic regression, with the regression coefficients representing the change in the probability of choice for each unit of change in the predictor.

### NPI features and perceived risk of transmission

Since in the analysis of the DCE data, we have multiple tests for each participant, Table 1 shows the False Discovery Rate LogWorth for each attribute, defined as − log10 (FDR *p*-value). The table also presents the FDR *p*-value for each attribute calculated using the Benjamini–Hochberg correction for multiple hypotheses testing^43^. This technique adjusts the p-values to correct the false discovery rate for multiple tests.

**Table 1:** Discrete choice experiment results models features (N = 6567).

Non-pharmaceutical intervention attribute	False discovery rate LogWorth	False discovery rate p-value	Likelihood-ratio chi-square	Degree of freedom	Prob > ChiSq
Face mask	3330.793	0.00000	6653.126	1	< 0.0001***
Duration	15.466	0.00000	66.546	1	< 0.0001***
Distance	59.285	0.00000	267.416	1	< 0.0001***
Environment	3557.559	0.00000	7107.195	1	< 0.0001***
People	149.709	0.00000	683.477	1	< 0.0001***

The marginal probabilities and utilities of each attribute are reported in Table 2. The results suggest that participants interpreted the attributes in the desired direction desired by health authorities. For example, wearing a face mask has an estimated negative utility of − 0.40, suggesting that a DCE scenario containing the wearing of a face mask was less likely to be selected and thus deemed as less risky compared to a scenario in which a face mask was not worn. By the same token, the attributes environment (i.e., indoors vs outdoors), the distance between people, duration of contact, and the number of people present were also pointing in the expected direction. Specifically, an outdoors setting had a negative utility of − 0.41, whereas adopting social distancing, i.e., being 2 m away from others, had a negative utility of − 0.08. Being in contact with two people instead of ten had a negative utility of − 0.13, while a short 15-min meeting (versus a 90-min one) had a smaller negative utility of − 0.04 (see Table 6 in the Supplementary Annex).

**Table 2:** Discrete choice experiment results for the main effect analysis of attributes, overall results (N = 6567).

Term	Estimate	Odds ratios	Percentage	Std error	Lower 95% of estimate	Upper 95% of estimate
Face mask [yes]	− 0.40387	0.67	− 33	0.00526	− 0.414211	− 0.393591
Duration [15 min]	− 0.04263	0.96	− 4	0.00524	− 0.052924	− 0.032371
Distance [about 1 m]	0.08525	1.09	+ 9	0.00524	0.0749876	0.0955421
Environment [outdoors, open air]	− 0.41644	0.66	− 34	0.00526	− 0.426779	− 0.406155
People [2 people]	− 0.13021	0.88	− 12	0.00501	− 0.140043	− 0.120397

AICc = 59,274.572; BIC = 59,318.917; − 2 × LogLikelihood = 59,264.571; − 2 × Firth LogLikelihood = 59,211.763.

The findings reported in Table 1 indicate that the two foremost attributes shaping participants’ assessment of the Sars-CoV-2 transmission risk are the environment and the use of face masks (see higher False Discovery Rate Log Worth). We converted the parameters of the logit regression into odds ratios and percentages to allow for a more straightforward interpretation. Meeting outdoors instead of indoors reduced the probability that respondents selected the transmission risk as the highest by 34%. Similarly, wearing a face mask rather than not wearing one reduced the probability by 33%. The other three NPI attributes were perceived as substantially less meaningful: The next most important attribute was the number of people involved in the contact. The presence of a small number of people^3^ instead of ten reduced the perceived transmission risk by 12%. The final two NPI attributes were least relevant for respondents’ judgement of the transmission risk, despite the prominent role that they have played in the media and public health campaigns or signage. Maintaining a social distance of 1 meter rather than 2 meters decreased the perceived transmission risk by just 9%. Finally, having a short 15 min meeting instead of a long 90-min one was the least influential attribute in participants’ evaluations, decreasing the judged transmission risk only by 4%.

### NPI features and perceived risk of transmission: cross-country comparisons

In the next step, we explore the cross-country variation in the perception of transmission risk (Table 3, Supplementary Annex 2). For this, we include the country of residence as an interaction term to identify whether the effect of the attribute *a*_*j*_ is smaller or larger in respondents from a specific country (odds ratio for the interaction term). Looking first at the two attributes with the largest weight, the effect of “wearing a face mask” is larger (reduces the perceived transmission risk more) in France, Italy and Spain, compared to Sweden, Bulgaria and Poland, while the effect of being outdoors is larger (reduces the perceived transmission risks to a larger extent) in Sweden, France, Spain and Bulgaria but less so in Italy. Turning to the attributes that have the least impact, social distancing (at 2 m) is considered more important in Spain, Sweden and Bulgaria, but less so in France or Italy, while meeting fewer people (two rather than ten people) has more relevance in France and Sweden and less in Italy, Spain, Bulgaria and Poland. The duration of a meeting has the same non-significant effect across all countries in our sample.

**Table 3:** Odds ratios conditional to the participants’ country, base percentage, country additional effect, and cumulative effect in brackets.

NPI attribute	Base percentages	Additional country effect in percentages					
		France	Italy	Spain	Sweden	Bulgaria	Poland
Face mask [yes]	− 34%***	− 10%*** (− 44%)	− 9%*** (− 43%)	− 19%*** (− 53%)	+ 12%*** (− 22%)	+ 23%*** (− 11%)	+ 9%*** (− 25%)
Duration [15 min]	− 5%***	+ 0.01%^n.s^	+ 0.02%^n.s.^	+ 0.001%^n.s.^	+ 0.01%^n.s.^	+ 0.0001%^n.s.^	− 0.01%^n.s.^
Distance [about 1 m]	+ 9%***	− 1%*** (+ 8%)	− 4%*** (+ 5%)	+ 1%*** (+ 10%)	+ 8%*** (+ 17%)	+ 3%*** (+ 12%)	− 0.2%*** (+ 9.2%)
Environment [outdoors, open air]	− 35%***	− 4%*** (− 39%)	+ 6%*** (− 29%)	− 3%*** (− 38%)	− 14%*** (− 49%)	+ 12% (− 22%)	+ 2%*** (− 33%)
People [2 people]	− 13%***	− 4%*** (− 17%)	+ 3%*** (− 10%)	+ 1%*** (− 12%)	− 7%*** (− 20%)	+ 5%*** (− 8%)	+ 2%*** (− 11%)

***p < 0.000, **p < 0.001, *p < 0.05, ^n.s^*p* > 0.05.

### NPI features and perceived risk of transmission: gender and age comparisons

Tables 4 and 5 show the effects of interaction terms that include participants’ gender and age. Notably, the two most important attributes (face masks and being outdoors) are even more important for women than men (full results in Supplementary Annex Sect. 3). The other attributes show some small differences but are less relevant than wearing face masks or being outdoors.

**Table 4:** Odds ratios conditional to the participants’ gender, base percentage, gender additional effect, and cumulative effect in brackets.

NPI attribute	Base percentages	Female	Male
Face mask [yes]	− 30%***	− 8%*** (− 38%)	− 2%*** (− 32%)
Duration [15 min]	+ 0.03%^n.s.^	+ 0.005%^n.s.^	− 0.007%^n.s.^
Distance [about 1 m]	+ 7%***	+ 3%*** (+ 10%)	− 0.005%^n.s.^ (+ 7%)
Environment [outdoors, open air]	− 28%***	− 13% (− 41%)	− 2%*** (− 30%)
People [2 people]	− 10%***	− 4%*** (− 14%)	− 1%*** (− 11%)

***p < 0.000, **p < 0.001, *p < 0.05, ^n.s.^*p* > 0.05.

**Table 5:** Odds ratios conditional to the participants’ age group, base percentage, age group additional effect, and cumulative effect in brackets.

NPI attribute	Base percentages	Age 18–24	Age 25–34	Age 35–44	Age 45–54	Age 55–64
Face mask [yes]	− 32%***	+ 1%*** (− 31%)	− 0.001%^n.s.^	− 3%*** (− 35%)	− 4%*** (− 28%)	+ 1%*** (− 31%)
Duration [15 min]	− 3%**	+ 0.03%^n.s.^	+ 0.02%^n.s.^	+ 0.001%^n.s.^	+ 0.02%^n.s.^	− 0.004%^n.s.^
Distance [about 1 m]	+ 8%***	+ 0.05%^n.s.^	− 0.001%^n.s.^	+ 0.001%^n.s.^	− 0.01%^n.s.^	+ 0.03%^n.s.^
Environment [outdoors, open air]	− 34%***	+ 10%*** (− 24%)	+ 4%*** (− 30%)	− 0.007%^n.s.^	− 4%*** (− 38%)	− 11%*** (− 45%)
People [2 people]	− 11%***	+ 0.03%^n.s.^	+ 0.009%^n.s.^	+ 0.001%^n.s.^	− 0.006%^n.s.^	+ 0.03%^n.s.^

***p < 0.000, **p < 0.001, *p < 0.05, ^n.s.^*p* > 0.05.

Concerning age (full results in Supplementary Annex Sect. 4), the most noticeable difference is the evaluation by the 18–24 years old group who do not perceive ‘being outdoors’ as decreasing the risk of transmission—in contrast to older age groups, particularly the 55–64 years old age group, who do.

#### NPI features and perceived risk of transmission: cognitive style and perceived fright

Next, the analysis investigates how cognitive style measured by the cognitive reflective test (CRT) and the ‘perceived fright’ of COVID-19 influence the weight of NPI attributes on transmission risk. First, we look at the conditional effects on the attributes’ odds ratios of two different scores of CRT tendency, the CRT reflective and CRT intuitive scores (see Tables 6, 7, full results are reported in Supplementary Annex Sect. 5).

**Table 6:** Odd ratio conditional to the cognitive reflective test-reflective score, base percentage, reflective score additional effect, and cumulative effect in brackets.

NPI attribute	Base percentages	Additional CRT effect in percentages
		CRT reflective
Face mask [yes]	− 31%***	− 3%*** (− 34%)
Duration [15 min]	− 3%**	− 1%** (− 4%)
Distance [about 1 m]	+ 6%***	+ 2%*** (+ 8%)
Environment [outdoors, open air]	− 27%***	− 6%*** (− 33%)
People [2 people]	− 9%***	− 3%*** (− 12%)

***p < 0.000, **p < 0.001, *p < 0.05, ^n.s.^*p* > 0.05.

**Table 7:** Odd ratio conditional to the cognitive reflective test-intuitive score, base percentage, intuitive score additional effect, and cumulative effect in brackets.

NPI attribute	Base percentages	Additional CRT effect in percentages
		CRT intuitive
Face mask [yes]	− 31%***	− 2%*** (− 33%)
Duration [15 min]	− 4%***	+ 0.001%^n.s.^
Distance [about 1 m]	+ 8%***	− 0.002%^n.s.^
Environment [outdoors, open air]	− 36%***	+ 1%* (− 35%)
People [2 people]	− 13%***	− 0.002%^n.s.^

***p < 0.000, **p < 0.001, *p < 0.05, ^n.s.^*p* > 0.05.

The CRT reflective score was computed following the procedure by Primi et al.^44^. The score distribution is reported in the Supplementary Annex Table. Respondents with high CRT *reflective* scores weigh each NPI attribute in the same direction as the main effect model. In other words, they added additional weight to or found a lower risk of transmission when the attributes pointed in the direction of the NPI (wearing a face mask, short meetings, few people, outdoors and with 2 m of social distance). Interestingly, participants with higher CRT reflective scores weighted the attribute ‘being outdoors’ as being more important than the others in lowering the perceived risk of transmission.

A strikingly contrasting picture appears in the model of the conditional effects on the attributes’ odds ratios of the CRT *intuitive* score. Only two interactions are statistically significant, namely the interactions between the CRT *intuitive* score and the attributes ‘face mask’ and ‘environment’ (see Table 6). Participants with higher CRT *intuitive* scores give even more weight to the transmission risk reduction in the presence of a face mask. However, they also give less weight to the effect of being outdoors on transmission risk reduction, compared to baseline.

A third model analyses the interaction between the NPI attributes and PFC (Perceived Fright of COVID-19), Table 8 (full results are reported in Supplementary Annex Sect. 6) reports the attributes’ odds ratios converted into probabilities and the odds ratio ratios of the four different levels of COVID-19 fear measured by PFC. For participants in the first level (who believe “COVID-19 is not frightening”), the effect of wearing a face mask on the perceived transmission risk reduction drops from − 32 to 3%. The change goes in the same direction but is much smaller for participants who are in level 2 of PFC, it drops from − 32 to − 27%). For respondents who express more fear of COVID-19 (levels 3 and 4), the presence of face masks, being outdoors or meeting with fewer people reduces the infection risks more than those who are less afraid of COVID-19 (PFC levels 1 and 2). For respondents with a very low level of PFC, wearing a face mask and being outdoors does little to reduce their perception of transmission risk.

**Table 8:** Odd ratio conditional to the level of perceived fright of covid (PFC), base percentage, PFC score additional effect, and cumulative effect in brackets.

Attribute	Base percentages	PFC level 1	PFC level 2	PFC level 3	PFC level 4
Face mask [yes]	− 32%***	+ 29%*** (− 3%)	+ 5%*** (− 27%)	− 3%*** (− 35%)	− 12%*** (− 44%)
Duration [15 min]	− 4%***	− 0.01%^n.s^	+ 0.003%^n.s^	+ 0.005%^n.s^	+ 0.004%^n.s^
Distance [about 1 m]	+ 9%***	+ 0.02%^n.s^	− 0.007%^n.s^	+ 0.002%^n.s^	− 0.02%^n.s^
Environment [outdoors, open air]	− 35%***	+ 8%*** (− 27%)	− 3%*** (− 38%)	+ 2%*** (− 33%)	− 4%*** (− 39%)
People [2 people]	− 12%***	+ 5%*** − 7%)	+ 0.004%^n.s^	− 1%*** (− 13%)	− 3%*** (− 15%)

***p < 0.000, **p < 0.001, *p < 0.05, ^n.s.^*p* > 0.05.

## Conclusions

The findings of this large DCE provide useful insights for improving public health guidance and increasing future resilience to pandemic events caused by novel transmissible viruses that need to be contained by NPIs. First, we reveal that participants assessed the perceived risks of SARS-Cov-2 transmissions based on two main NPI attributes: wearing face masks and meeting outdoors. Other NPIs that featured prominently in public health communications were largely ignored, including social distancing, the meeting duration and the number of people at a meeting. Analyses of the effects by country, gender, and age identified some differences between subgroups within the sample but overall confirmed the general finding that some NPI attributes are rarely taken into consideration and have only a small impact on the perceived reduction of the risk of transmission. These findings imply that people do not, or cannot, consider all of the facets that are simultaneously present in a real-life scenario. These findings align with the behavioural insight that people reduce or ‘substitute’ complex multidimensional contexts with relatively simple heuristics^45,46^.

Our study was based on the combination of national samples and a discrete choice online experiment, and it suffers from the limitations associated with these ways of collecting data. The DCE was a simulated scenario, and while we aimed at the highest ecological validity possible, it remained a stylized version of reality in terms of decision-making. That said, DCEs have been used successfully to elicit health preferences^47^, and findings from this experimental method appear to be internally, ecologically and externally valid. The unique opportunity of carrying out our DCE on several national samples of unvaccinated citizens during the peak of the first phase of the pandemic adds further value to our study.

These findings have two implications for public health policy. Firstly, policymakers and designers of information campaigns should align the communication of NPIs to the public’s *perception* of how these NPIs lower the transmission risk (in addition to taking into account their actual epidemiological importance for the virus transmission). This is all the more important for the NPIs that are universally considered most important: wearing a face mask or being outdoors. For instance, while being outdoors objectively reduced the risk of transmission^48,49^, and most of our participants made their decisions in the DCE accordingly, this was less so the case for younger participants. This suggests that the communication of the impact of being outdoors versus indoors has to be communicated differently to young adults who need and desire to be indoors more than other age groups.

Interestingly, respondents considered the importance of social distancing for reducing transmission risk as less pronounced, even though it was widely promoted as an important measure, along with the use of face masks and with being outdoors. The underlying reasons for the different evaluation by respondents in this study needs further investigation, but regardless, we can infer a second course recommendation for health policymakers: “boost” the appreciation and improve communication on NPI attributes that the public generally ignores. NPIs about social distancing, the number of people to meet, and the duration of a meeting were generally given little weight in the assessment of transmission risk and, therefore, do not reduce the perceived risk of transmission as much as they could and should. Notably, social distancing and other neglected NPIs are the very measures featured in the recommended safety guidelines of most countries around the world. Raising awareness about the safety-related behaviours that are less prominent in people’s risk assessment is a possible behavioural intervention. Examples of making neglected criteria and behaviours more salient include reminders to pay attention to the aspects that are often ‘naturally’ neglected such as promoting physical distancing through floor/pavement markings or through signs on chairs and benches to leave space between one person and the next. Overall, the goal would be to help citizens to apply a multi-attributes risk assessment rather than relying on just one or two criteria.

The heterogeneity analysis by cognitive style shows that there are differences in the perception of transmission risk and of the role of the different attributes between more heuristic-prone individuals (i.e., those with higher scores in the intuitive CRT test) and those with high reflective CRT scores which are more inclined to systematic thinking. Most people were in the former group and assessed the transmission risk based on one or two aspects only (usually face masks and being outdoor/indoor). In contrast, only a minority of respondents were capable of using several NPI attributes in their evaluations. A possible policy lesson of this finding could be to advertise packages of NPI measures in a simplified format to reduce cognitive load. One example could be the combined “AHA” rule applied in Germany, which was used as an abbreviation for (1) distance (“Abstand”), (2) hygiene (“Hygiene”), and (3) wearing masks (“Atemschutz”).

The broader message from this study is that people’s understanding of complex transmission risk models is generally simple, and this should be reflected in developing policy interventions. Because of bounded cognitive ability^50^, complex multidimensional scenarios cannot be absorbed, processed and acted upon with perfect accuracy. This is true in an online choice experiment with stylised constellations of NPI features and even more true in complex real-life situations where the social pressure to remove or lower face masks below one’s nose, move a step closer to another person, go out or stay indoors are all simultaneously present in our mind and in our social environment.

Not knowing the distribution of ‘systematic thinkers’ in one’s population, it is reasonable to expect that most of the public confronting a novel virus with a high transmission risk will use a small amount of the publicly available information when making their judgments about transmission risk. As a result, the recommended course of action is to ensure that campaigns include the minimum amount of epidemiologically important criteria. Other criteria (e.g., number of people involved), which lower transmission risks but are naturally weighed less, should be addressed by ‘distributed cognition’^51^. This involves using choice architecture to reduce the cognitive burden when the probability of a multi-criteria risk assessment by citizens is low. This study contributes to establishing a realistic picture of people’s decision-making processes and behaviours in the context of a pandemic. Further aspects of the decision-making process, such as the role of cognitive scarcity^52^ and the underlying reasons for discrepancies in risk assessments require further research.

## Methods

### Overall data collection (sample and procedure)

Online surveys were conducted in six European countries, namely Bulgaria (*n* = 1069), France (*n* = 1108), Poland (*n* = 1104), Italy (*n* = 1087), Spain (*n* = 1102) and Sweden (*n* = 1097). The surveys were fielded in June 2021. In each country, the sample comprised unvaccinated respondents aged 18 years and older in the participant pool of the survey company Respondi. The survey experiment was carried out according to the current EU regulations. The Ethical Committee of the University of Trento approved the experimental protocol. All participants gave their informed consent.

A quota sample was selected to match the census population of each target country in terms of (1) gender, (2) age, (3) education level, and (4) region or state within each country. The online survey took between 20 and 30 min (average 24 min) to complete and included the DCE, the sociodemographic information. The expanded version of the Cognitive Reflective Test (CRT)^43,53^ that distinguishes between those with low or high reflective tendency and low or high intuitive tendency.

Participants’ Perceived Fright of COVID-19 (PFC) was measured as a 5-level variable with a value of 1 indicating lowest fear and a value of 5 indicating highest fear, with 5 taken as the baseline. No personally identifying information was collected, and the data was fully anonymised. Upon completing the survey, participants received vouchers worth between three and five Euros, depending on the countries’ income levels.

### Discrete choice experiment

The DCE method goes beyond both traditional qualitative assessments of risk perception and quantitative ranking and evaluation surveys, which do not provide information on the strength of preferences and the trade-offs or the likelihood of choosing one course of action over another^46^. An advantage of the DCE method instead involves the respondent in making a series of discrete choices, requiring trade-offs between different combinations of attributes, in this case, transmission NPIs, which allows the researcher to determine the relative importance of each NPI feature.

The DCE methodology is based on random utility maximization theory, which assumes that individuals choose the utility-maximizing option when presented with a choice set containing alternative scenarios.

In our DCE, respondents were presented with 8 binary choices (16 items in total) employed to estimate the relative risk of COVID-19 transmission for each of the five NPIs attributes. Specifically, the choice sets varied with respect to the following attributes: (a) personal protective equipment: mask/no mask, (b) environment: indoors vs outdoors, (c) duration of contact: short (15 min) vs long (90 min), (d) the number of people: few (2 people) vs many (10 people) and (e) physical distance: short (1 m) vs long (2 m). Respondents were presented with pairs of mutually exclusive combinations of these public health guidelines attributes and asked to select which one out of two (hypothetical) scenarios they perceived as riskier regarding SARS-Cov-2 transmission (see Table 9 for an example of one of the 8 choice sets). Supplementary Annex Sections 3–5 report the models and additional details for the DCE analysis.

**Table 9:** Examples of the DCE transmission risk scenarios.

NPI attributes	Scenario 1	Scenario 2
Mask wearing	With mask	Without mask
Environment	Outdoors	Indoors
Contact duration	90 min	15 min
Number of people	10 people	2 people
Physical distance	1 m apart	2 m apart

The analysis also allowed us to investigate whether participants engage in a multi-attribute evaluation of all the available information in each scenario or simplify the task with a heuristic basing their judgements on a few attributes only. When the experiment was carried out, at that specific stage of the COVID-19 pandemic, we did not have any prior information about any differential weight of NPI attributes, therefore, we designed a neutral utility design using an orthogonal d-optimal design^54^.

Because our primary interest lies in comparing groups rather than individuals, as they are the target of potential public health policy interventions, we performed a conditional logistic regression rather than a random parameters logit. We used a conditional logit regression, as stated in the paper:

$$P\left(i|j\right)=\frac{{e}^{{\beta}{\prime}{X}_{ij}}}{{\sum }_{k\epsilon c}{e}^{{\beta}{\prime}{X}_{ik}}},$$

where $P\left(i|j\right)$ is the probability that individual *i* chooses alternative j, ${X}_{ij}$ is a vector of observed variables for alternative *j* and individual *i*, $\beta$ is a vector of coefficients to be estimated, *e* is the base of the natural logarithm, and the denominator ${\sum }_{k\epsilon c}{e}^{{\beta}{\prime}{X}_{ik}}$ is the sum of the exponentiated utilities of all available alternatives in the choice set *C*.

### Supplementary Information

Supplementary Information.

## Data Availability

All data generated or analysed during this study are made publicly available via the Open Science Framework under the following link: https://osf.io/72jrq/.
