# Supplementary material for: Assessing the perceived effect of non-pharmaceutical interventions on SARS-Cov-2 transmission risk: an experimental study in Europe
Source: Sci Rep. 2024 Feb 28;14:4857. doi: 10.1038/s41598-024-55447-1 (PMC10902314; doi:10.1038/s41598-024-55447-1)
Supplement: Supplementary file 1 — Supplementary Information. [file 41598_2024_55447_MOESM1_ESM.docx]

# **Annex**

Index

[**Annex** 1](#_Toc158036933)

[1. Additional information on the DCE and descriptive statistics of main variables 2](#_Toc158036934)

[1.1 Distribution of our sample 4](#_Toc158036935)

[1.2 CRT scores distributions 5](#_Toc158036936)

[1.3 CRT distribution across countries 8](#_Toc158036937)

[1.4 PERCEIVED FRIGHT OF COVID-19 (PFC) 10](#_Toc158036938)

[1.5 Discrete Choice Experiment 11](#_Toc158036939)

[2. DCE Countries differences 12](#_Toc158036940)

[3. DCE Gender interaction term model 14](#_Toc158036941)

[4. DCE Age interaction 16](#_Toc158036942)

[5. DCE CRT Models 18](#_Toc158036943)

[6. DCE Interaction with perceived fright of COVID19 (PFC) 20](#_Toc158036944)

**Index of Tables**

[Supplementary Table 1 Quantiles of the Cognitive Reflective Test-Reflective Score 6](#_Toc158036945)

[Supplementary Table 2 Summary statistics for Cognitive Reflective Test-Reflective Score 7](#_Toc158036946)

[Supplementary Table 3 Quantiles of the Cognitive Reflective Test-Intuitive Score 8](#_Toc158036947)

[Supplementary Table 4 Summary statistics for Cognitive Reflective Test-Intuitive Score 8](#_Toc158036948)

[Supplementary Table 5 CRT Reflective score mean values by countries. 9](#_Toc158036949)

[Supplementary Table 6 CRT Intuitive score mean scores by countries. 10](#_Toc158036950)

[Supplementary Table 7 Perceived fright score quartiles. 11](#_Toc158036951)

[Supplementary Table 8 Mean and Std Dev of the perceived fright indicator in the overall sample. 11](#_Toc158036952)

[Supplementary Table 9 Full list of choice sets for the DCE experiment 12](#_Toc158036953)

[Supplementary Table 10 Marginal probabilities and utilities of each attribute value. 12](#_Toc158036954)

[Supplementary Table 11 Odds ratios country effect 13](#_Toc158036955)

[Supplementary Table 12 Choice model main effect and country interaction, effect summary, parameter estimates and likelihood ratio tests 13](#_Toc158036956)

[Supplementary Table 13 Choice model main effect and gender interaction, effect summary, parameter estimates and likelihood ratio tests 15](#_Toc158036957)

[Supplementary Table 14 Choice model main effect and age group interaction, effect summary, parameter estimates and likelihood ratio tests 17](#_Toc158036958)

[Supplementary Table 15 Choice model main effect and CRT Reflective score interaction, effect summary, parameter estimates and likelihood ratio tests 19](#_Toc158036959)

[Supplementary Table 16 Choice model main effect and CRT-Intuitive score interaction, effect summary, parameter estimates and likelihood ratio tests 20](#_Toc158036960)

[Supplementary Table 17 Choice model main effect and Perceived Fright of Covid19 score (PFC) interaction, effect summary, parameter estimates and likelihood ratio tests 21](#_Toc158036961)

**Index of Figures**

[Supplementary Figure 1 Example of choice card from the DCE implemented online. 3](#_Toc158036962)

[Supplementary Figure 2 Distributions for the country, age, gender, and education level 5](#_Toc158036963)

[Supplementary Figure 3 Distribution fit for Cognitive Reflective Test-Reflective Score. 6](#_Toc158036964)

[Supplementary Figure 4 Distribution fit for Cognitive Reflective Test-Intuitive Score. 7](#_Toc158036965)

[Supplementary Figure 5 CRT Reflective score means across countries. 9](#_Toc158036966)

[Supplementary Figure 6 CRT Intuitive Score means across countries. 10](#_Toc158036967)

# Additional information on the DCE and descriptive statistics of main variables

Example of choice card from the DCE implemented online

Supplementary Figure 1 Example of choice card from the DCE implemented online.


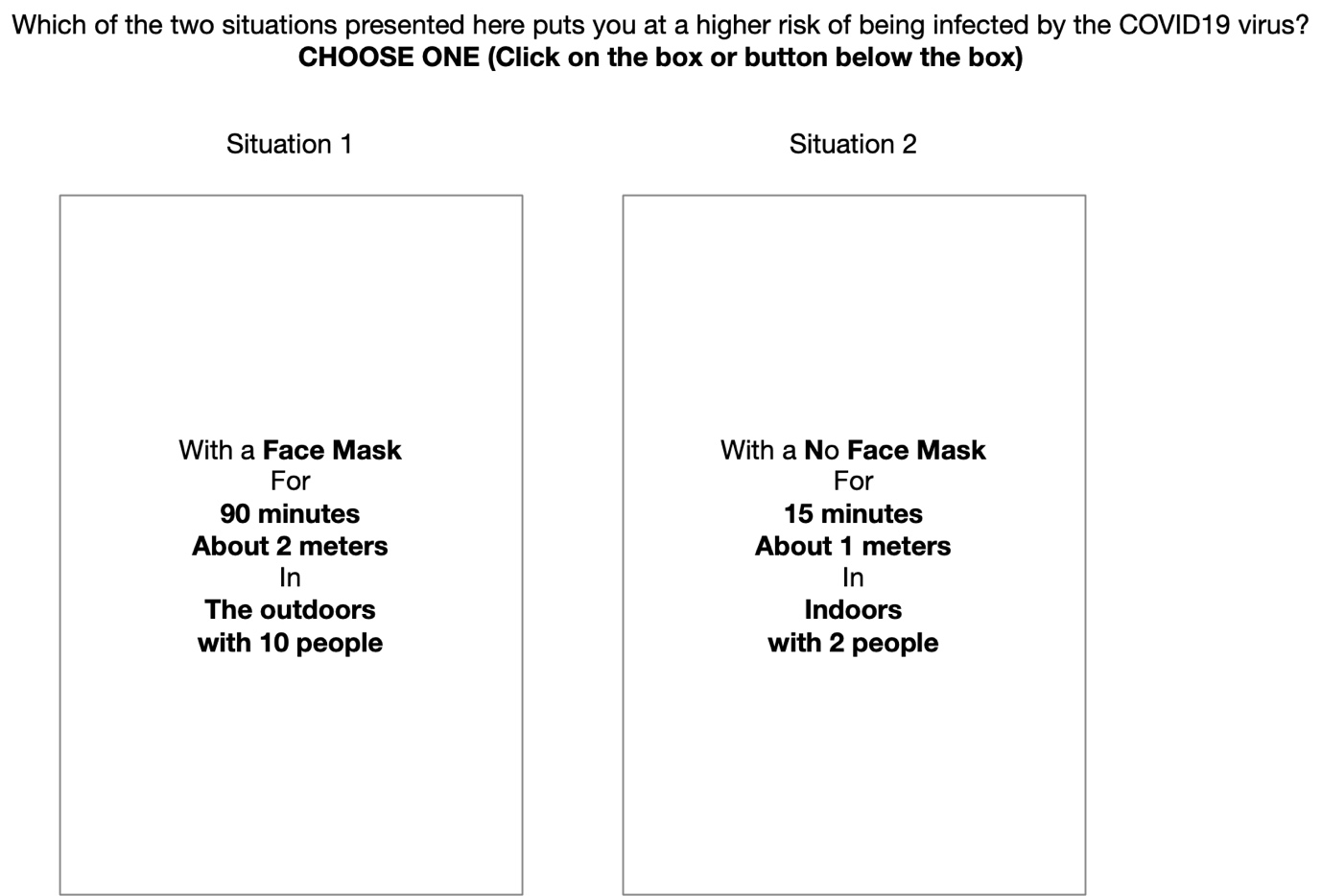


Ngene syntax to create the DCE:

Design

;alts= A, B

;rows= 8

;orth=ood

;model:

U(A)= b1+ b2 * FM[0,1] + b3 * Duration[0,1]+ b4 * Distance[0,1]+ b5 * Env[0,1]+ b5 * People[0,1]/

U(B)= b2 * FM + b3 * Duration + b4 * Distance + b5 * Env + b5 * People

$

Ngene design information:

## 1.1 Distribution of our sample

Supplementary Figure 2 Distributions for the country, age, gender, and education level


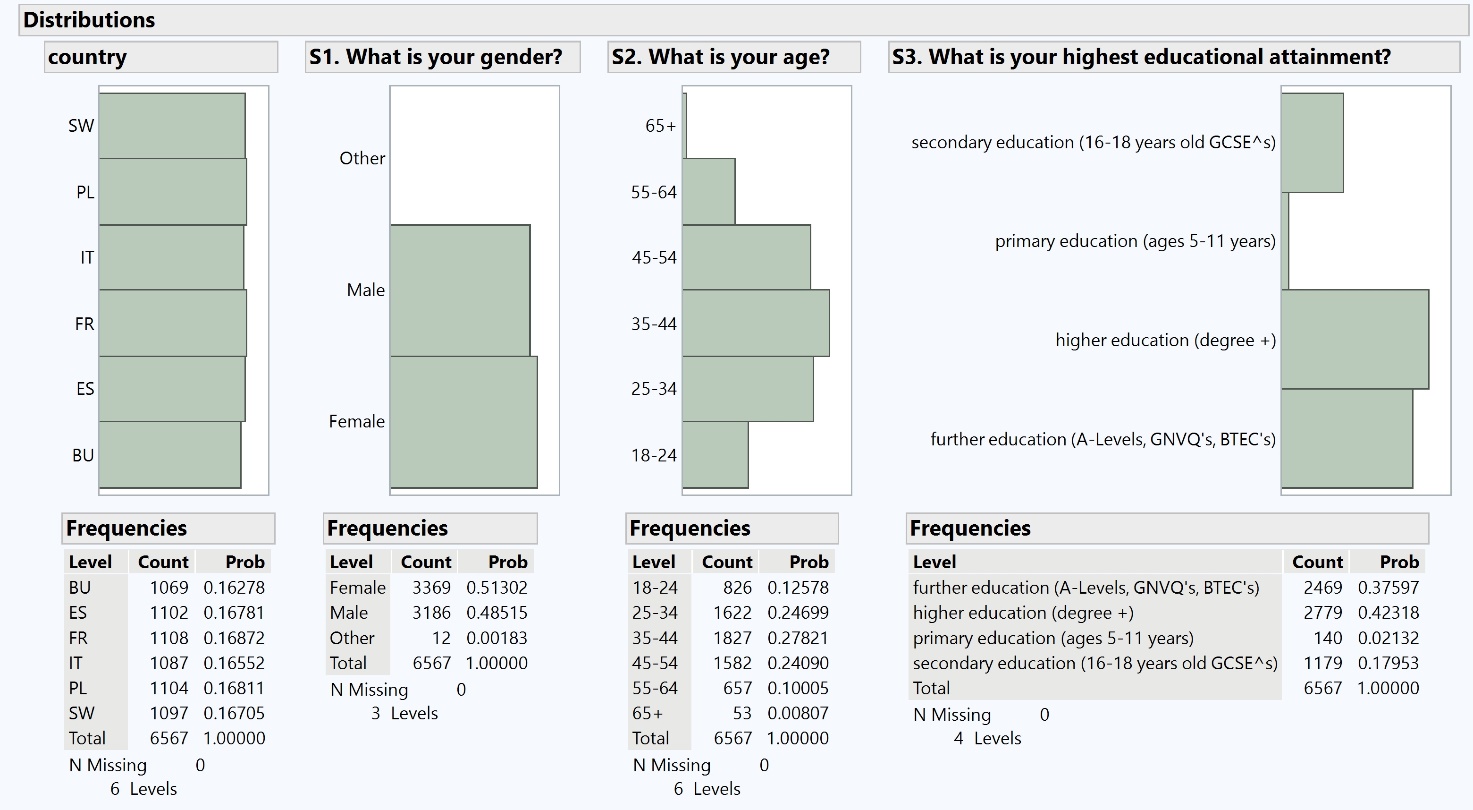


## 1.2 CRT scores distributions

**Reflective score (CRT)**

Supplementary Figure 3 Distribution fit for Cognitive Reflective Test-Reflective Score.


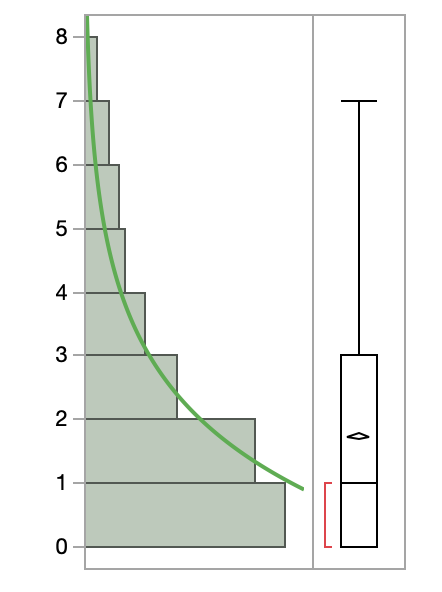


Quantiles

Supplementary Table 1 Quantiles of the Cognitive Reflective Test-Reflective Score

| 100,0% | maximum | 7 |
| --- | --- | --- |
| 99,5% |  | 7 |
| 97,5% |  | 6 |
| 90,0% |  | 5 |
| 75,0% | quartile | 3 |
| 50,0% | median | 1 |
| 25,0% | quartile | 0 |
| 10,0% |  | 0 |
| 2,5% |  | 0 |
| 0,5% |  | 0 |
| 0,0% | minimum | 0 |

Summary Statistics

Supplementary Table 2 Summary statistics for Cognitive Reflective Test-Reflective Score

| Mean | 1,7348866 |
| --- | --- |
| Std Dev | 1,8424644 |
| Std Err Mean | 0,0227361 |
| Upper 95% Mean | 1,7794567 |
| Lower 95% Mean | 1,6903164 |
| N | 6567 |

Intuitive score (CRT)

Supplementary Figure 4 Distribution fit for Cognitive Reflective Test-Intuitive Score.


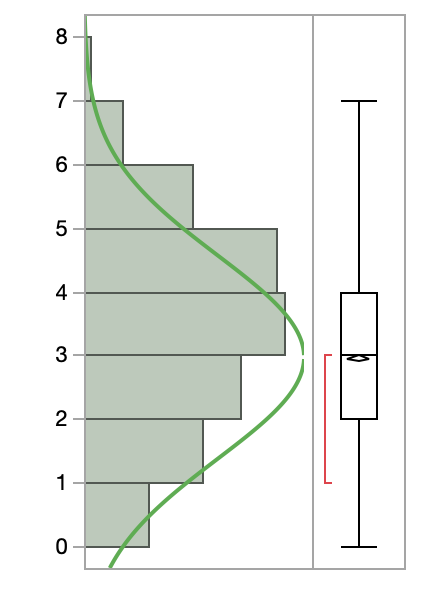


Supplementary Table 3 Quantiles of the Cognitive Reflective Test-Intuitive Score

| 100,0% | maximum | 7 |
| --- | --- | --- |
| 99,5% |  | 7 |
| 97,5% |  | 6 |
| 90,0% |  | 5 |
| 75,0% | quartile | 4 |
| 50,0% | median | 3 |
| 25,0% | quartile | 2 |
| 10,0% |  | 1 |
| 2,5% |  | 0 |
| 0,5% |  | 0 |
| 0,0% | minimum | 0 |

Supplementary Table 4 Summary statistics for Cognitive Reflective Test-Intuitive Score

| Mean | 2,9616263 |
| --- | --- |
| Std Dev | 1,6002555 |
| Std Err Mean | 0,0197472 |
| Upper 95% Mean | 3,0003373 |
| Lower 95% Mean | 2,9229153 |
| N | 6567 |

## 1.3 CRT distribution across countries

***CRT Reflective Score***

Supplementary Figure 5 CRT Reflective score means across countries.


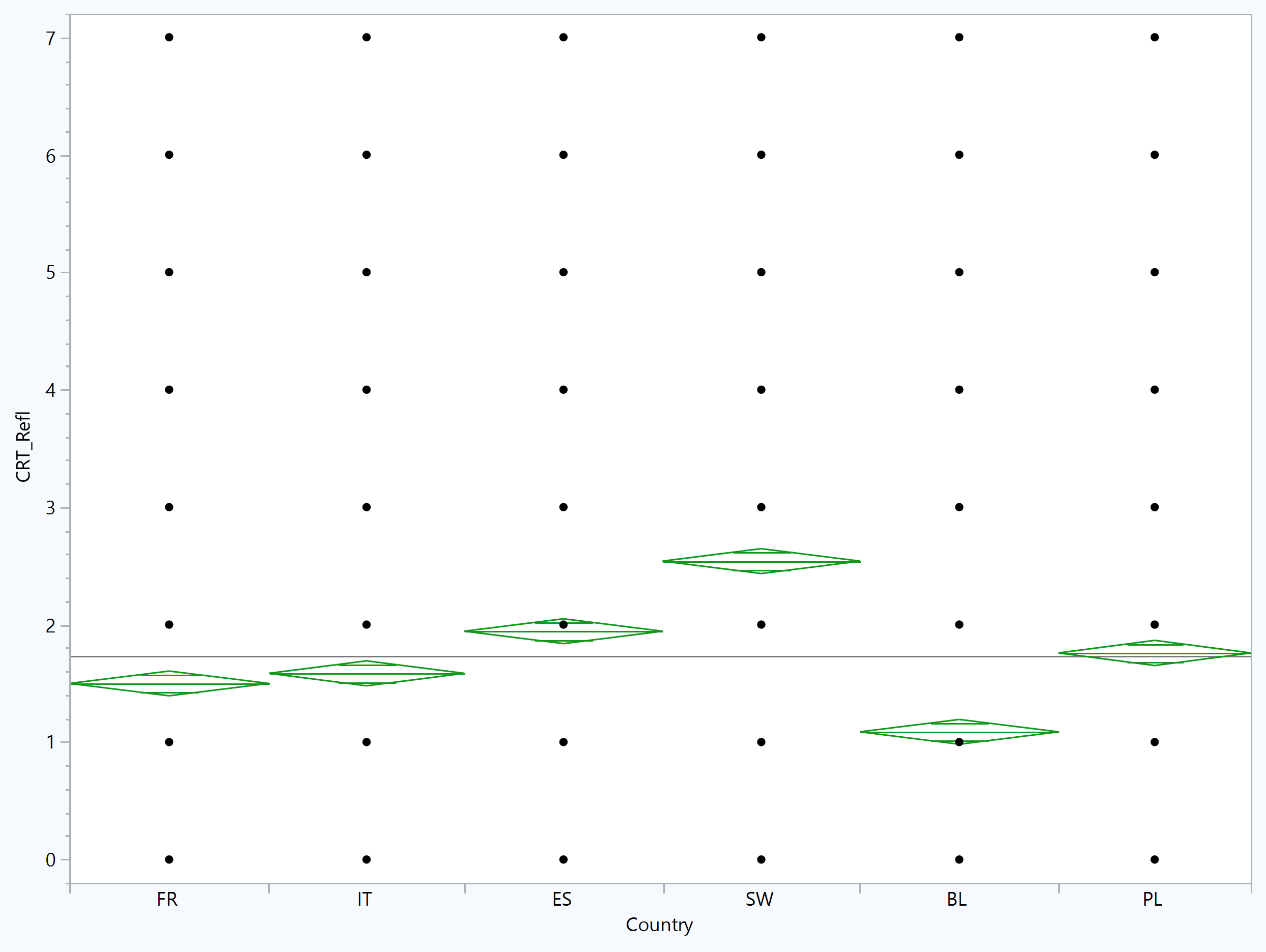


Supplementary Table 5 CRT Reflective score mean values by countries.

| **Country** | ***N*** | **Mean** | **Std Error** |
| --- | --- | --- | --- |
| FR | 1108 | 1.49910 | 0.05372 |
| IT | 1087 | 1.58510 | 0.05424 |
| ES | 1102 | 1.94374 | 0.05387 |
| SW | 1097 | 2.54057 | 0.05399 |
| BL | 1104 | 1.08696 | 0.05382 |
| PL | 1069 | 1.75865 | 0.05469 |

*CRT Intuitive Score*

Supplementary Figure 6 CRT Intuitive Score means across countries.


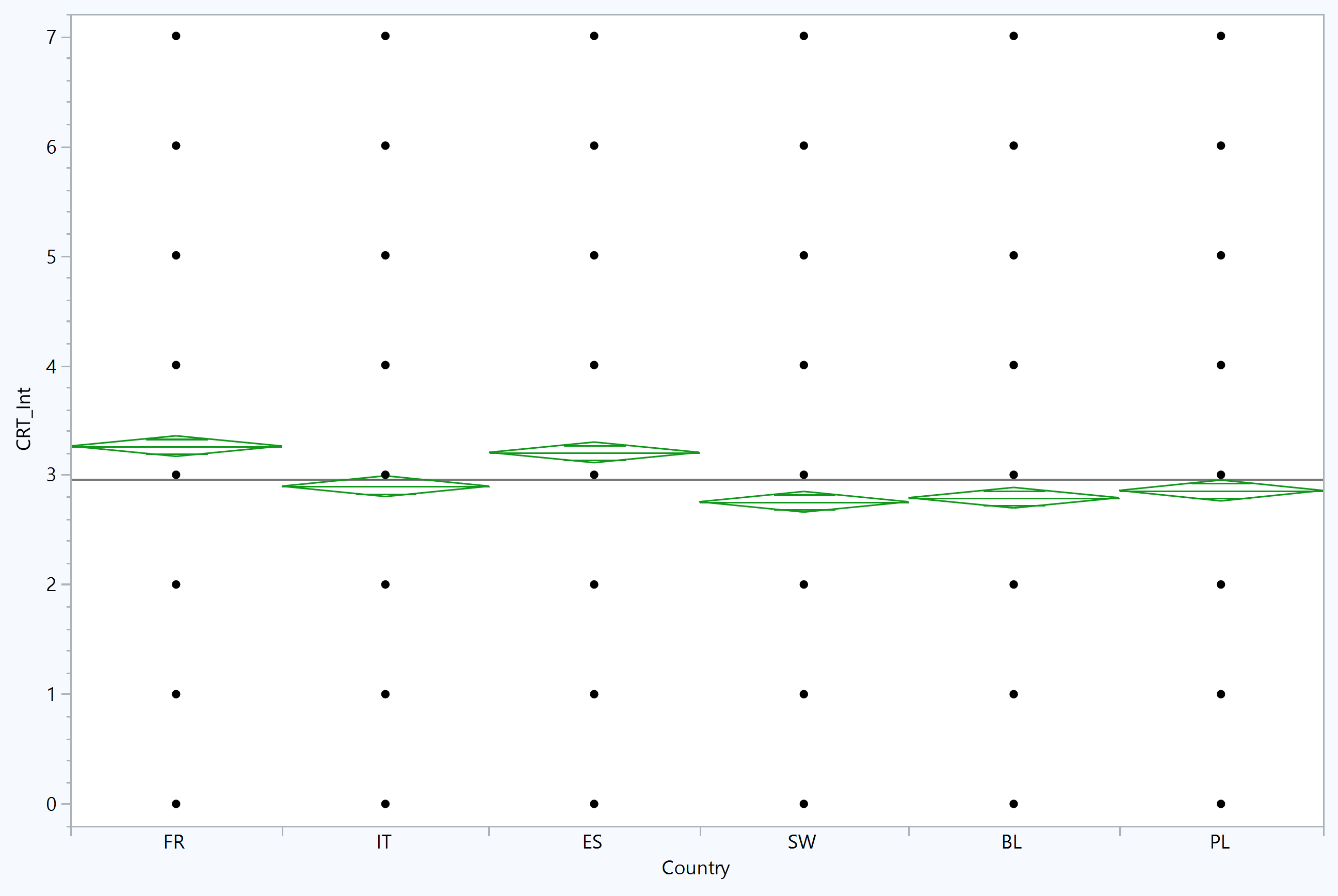


Supplementary Table 6 CRT Intuitive score mean scores by countries.

| **Country** | **N** | **Mean** | **Std Error** |
| --- | --- | --- | --- |
| FR | 1108 | 3.26173 | 0.04772 |
| IT | 1087 | 2.89604 | 0.04818 |
| ES | 1102 | 3.20417 | 0.04785 |
| SW | 1097 | 2.75387 | 0.04796 |
| BL | 1104 | 2.79076 | 0.04781 |
| PL | 1069 | 2.85688 | 0.04858 |

## 1.4 PERCEIVED FRIGHT OF COVID-19 (PFC)

Supplementary Table 7 Perceived fright score quartiles.

| 100,0% | maximum | 5 |
| --- | --- | --- |
| 99,5% |  | 5 |
| 97,5% |  | 5 |
| 90,0% |  | 5 |
| 75,0% | quartile | 4 |
| 50,0% | median | 3 |
| 25,0% | quartile | 2 |
| 10,0% |  | 1 |
| 2,5% |  | 1 |
| 0,5% |  | 1 |
| 0,0% | minimum | 1 |

Supplementary Table 8 Mean and Std Dev of the perceived fright indicator in the overall sample.

| Mean | 3,1997868 |
| --- | --- |
| Std Dev | 1,2451477 |
| Std Err Mean | 0,0153652 |
| Upper 95% Mean | 3,2299075 |
| Lower 95% Mean | 3,1696661 |
| N | 6567 |

## 1.5 Discrete Choice Experiment

Supplementary Table 9 Full list of choice sets for the DCE experiment

| **Choice Set** | **Face Mask** | **Duration** | **Distance** | **Environment** | **People** |
| --- | --- | --- | --- | --- | --- |
| 1 | No | 90 minutes | About 1 meter | Indoors, closed room | 10 people |
| 1 | Yes | 15 minutes | About two meters | Outdoors, open air | 2 people |
| 2 | Yes | 90 minutes | About two meters | Indoors, closed room | 2 people |
| 2 | No | 15 minutes | About 1 meter | Outdoors, open air | 10 people |
| 3 | No | 90 minutes | About 1 meter | Indoors, closed room | 2 people |
| 3 | Yes | 15 minutes | About two meters | Outdoors, open air | 10 people |
| 4 | Yes | 90 minutes | About 1 meter | Outdoors, open air | 10 people |
| 4 | No | 15 minutes | About two meters | Indoors, closed room | 2 people |
| 5 | Yes | 15 minutes | About 1 meter | Indoors, closed room | 2 people |
| 5 | No | 90 minutes | About two meters | Outdoors, open air | 10 people |
| 6 | No | 90 minutes | About two meters | Outdoors, open air | 2 people |
| 6 | Yes | 15 minutes | About 1 meter | Indoors, closed room | 10 people |
| 7 | Yes | 90 minutes | About 1 meter | Outdoors, open air | 2 people |
| 7 | No | 15 minutes | About two meters | Indoors, closed room | 10 people |
| 8 | No | 15 minutes | About 1 meter | Outdoors, open air | 2 people |
| 8 | Yes | 90 minutes | About two meters | Indoors, closed room | 10 people |

Supplementary Table 10 Marginal probabilities and utilities of each attribute value.

| **Marginal Probability** | **Marginal Utility** | **Face Mask** |
| --- | --- | --- |
| 0,3084 | -0,40388 | Yes |
| 0,6916 | 0,40388 | No |

| **Marginal Probability** | **Marginal Utility** | **Duration** |
| --- | --- | --- |
| 0,4787 | -0,04264 | 15 minutes |
| 0,5213 | 0,04264 | 90 minutes |

| **Marginal Probability** | **Marginal Utility** | **Distance** |
| --- | --- | --- |
| 0,5425 | 0,08526 | About 1 meter |
| 0,4575 | -0,08526 | About two meters |

| **Marginal Probability** | **Marginal Utility** | **Environment** |
| --- | --- | --- |
| 0,3030 | -0,41645 | Outdoors, open air |
| 0,6970 | 0,41645 | Indoors, closed room |

| **Marginal Probability** | **Marginal Utility** | **People** |
| --- | --- | --- |
| 0,4353 | -0,13021 | 2 people |
| 0,5647 | 0,13021 | 10 people |

# 2. DCE Countries differences

There are countries' differences in the baseline DCE, in terms of the relative importance of attributes. See below a brief reporting:

Supplementary Table 11 Odds ratios country effect

|  |  | **Additional country odds ratios effect** | | | | | |
| --- | --- | --- | --- | --- | --- | --- | --- |
| **Attribute** | **Base odds ratio** | **FR** | **IT** | **ES** | **SW** | **BL** | **PL** |
| Face Mask [Yes] | 0.66 | 0.90 | 0.91 | 0.81 | 1.12 | 1.23 | 1.09 |
| Duration [15 minutes] | 0.95 | 0.98 | n.s | n.s | n.s | n.s | 0.99 |
| Distance [About 1 meter] | 1.09 | 0.99 | 0.96 | 1.01 | 1.08 | 0.97 | 0.99 |
| Environment [Outdoors, open air] | 0.65 | 0.96 | 1.06 | 0.97 | 0.86 | 1.12 | 1.02 |
| People [2 people] | 0.87 | 0.96 | 1.03 | 1.01 | 0.93 | 1.05 | 1.02 |

Choice Model: Main effect + Country interaction

Supplementary Table 12 Choice model main effect and country interaction, effect summary, parameter estimates and likelihood ratio tests

| **Source** | **FDR LogWorth** | **FDR PValue** |
| --- | --- | --- |
| Environment | 3637.650 | 0.00000 |
| Face Mask | 3378.338 | 0.00000 |
| country*Face Mask | 163.052 | 0.00000 |
| People | 155.743 | 0.00000 |
| Distance | 60.532 | 0.00000 |
| country*Environment | 44.778 | 0.00000 |
| Duration | 15.706 | 0.00000 |
| country*People | 13.878 | 0.00000 |
| country*Distance | 9.385 | 0.00000 |
| country*Duration | 0.565 | 0.27223 |

**Parameter Estimates**

| **Term** | **Estimate** | | **Std Error** | | **Lower 95%** | | **Upper 95%** |  |
| --- | --- | --- | --- | --- | --- | --- | --- | --- |
| Face Mask[Yes] | -0.415385378 | | 0.0054296395 | | -0.426052 | | -0.404772 |  |
| Duration[15 minutes] | -0.044472254 | | 0.0054080543 | | -0.055081 | | -0.033886 |  |
| Distance[About 1 meter] | 0.088827991 | | 0.0054090689 | | 0.0782406 | | 0.0994398 |  |
| Environment[Outdoors, open air] | -0.429817120 | | 0.0054357265 | | -0.440496 | | -0.419192 |  |
| People[2 people] | -0.134952128 | | 0.0050963517 | | -0.144946 | | -0.124972 |  |
| country[FR]*Face Mask[Yes] | -0.103865048 | | 0.0126152297 | | -0.128723 | | -0.079276 |  |
| country[FR]*Duration[15 minutes] | -0.011301165 | | 0.0125244399 | | -0.035905 | | 0.0131881 |  |
| country[FR]*Distance[About 1 meter] | -0.004611764 | | 0.0125257334 | | -0.029102 | | 0.0199965 |  |
| country[FR]*Environment[Outdoors, open air] | -0.039592455 | | 0.0125985901 | | -0.064413 | | -0.015032 |  |
| country[FR]*People[2 people] | -0.041039418 | | 0.0116193254 | | -0.063848 | | -0.018305 |  |
| country[IT]*Face Mask[Yes] | -0.093460477 | | 0.0121051023 | | -0.117281 | | -0.069837 |  |
| country[IT]*Duration[15 minutes] | 0.028036685 | | 0.0120607137 | | 0.0043836 | | 0.0516557 |  |
| country[IT]*Distance[About 1 meter] | -0.043984843 | | 0.0120613354 | | -0.067603 | | -0.020329 |  |
| country[IT]*Environment[Outdoors, open air] | 0.056728154 | | 0.0120890976 | | 0.0329499 | | 0.0803316 |  |
| country[IT]*People[2 people] | 0.027687424 | | 0.0114398933 | | 0.0052461 | | 0.0500851 |  |
| country[ES]*Face Mask[Yes] | -0.213555523 | | 0.0132949272 | | -0.2398 | | -0.187686 |  |
| country[ES]*Duration[15 minutes] | -0.002463725 | | 0.0132304274 | | -0.028482 | | 0.0233839 |  |
| country[ES]*Distance[About 1 meter] | 0.010079644 | | 0.0132314936 | | -0.015767 | | 0.0361033 |  |
| country[ES]*Environment[Outdoors, open air] | -0.034447785 | | 0.0132657806 | | -0.060624 | | -0.008624 |  |
| country[ES]*People[2 people] | 0.014618236 | | 0.0118618000 | | -0.008657 | | 0.0378377 |  |
| country[SW]*Face Mask[Yes] | 0.112267421 | | 0.0122245451 | | 0.0882194 | | 0.1361337 |  |
| country[SW]*Duration[15 minutes] | -0.014363950 | | 0.0121804970 | | -0.038293 | | 0.009449 |  |
| country[SW]*Distance[About 1 meter] | 0.075242842 | | 0.0121893043 | | 0.0514235 | | 0.0992003 |  |
| country[SW]*Environment[Outdoors, open air] | -0.121251031 | | 0.0123492703 | | -0.14557 | | -0.097167 |  |
| country[SW]*People[2 people] | -0.076015091 | | 0.0116385463 | | -0.098872 | | -0.053253 |  |
| country[BL]*Face Mask[Yes] | 0.207541342 | | 0.0108421788 | | 0.1862718 | | 0.2287632 |  |
| country[BL]*Duration[15 minutes] | 0.000144472 | | 0.0108274432 | | -0.021081 | | 0.0213531 |  |
| country[BL]*Distance[About 1 meter] | -0.034361629 | | 0.0108280408 | | -0.055571 | | -0.013135 |  |
| country[BL]*Environment[Outdoors, open air] | 0.118538329 | | 0.0108510257 | | 0.0972426 | | 0.1397685 |  |
| country[BL]*People[2 people] | 0.048348161 | | 0.0106063308 | | 0.0275528 | | 0.0691214 |  |
| country[PL]*Face Mask[Yes] | | 0,091072285 | | 0,0116191476 | | 0,0682413 | | 0,1137794 |
| country[PL]*Duration[15 minutes] | | -0,000052318 | | 0,0115951229 | | -0,0228 | | 0,0226447 |
| country[PL]*Distance[About 1 meter] | | -0,002364250 | | 0,0115962479 | | -0,02506 | | 0,0203891 |
| country[PL]*Environment[Outdoors, open air] | | 0,020024789 | | 0,0116324005 | | -0,002839 | | 0,0427505 |
| country[PL]*People[2 people] | | 0,026400688 | | 0,0111647581 | | 0,0045023 | | 0,0482611 |

| AICc | 58190.715 |
| --- | --- |
| BIC | 58456.757 |
| -2*LogLikelihood | 58130.679 |
| -2*Firth LogLikelihood | 57850.728 |

Converged in Gradient

Firth Bias-Adjusted Estimates

Likelihood Ratio Tests

| **Source** | **L-R ChiSquare** | **DF** | **Prob>ChiSq** |
| --- | --- | --- | --- |
| Face Mask | 6748.805 | 1 | <.0001* |
| Duration | 68.337 | 1 | <.0001* |
| Distance | 274.075 | 1 | <.0001* |
| Environment | 7267.957 | 1 | <.0001* |
| People | 712.034 | 1 | <.0001* |
| country*Face Mask | 770.590 | 5 | <.0001* |
| country*Duration | 6.366 | 5 | 0.2722 |
| country*Distance | 52.791 | 5 | <.0001* |
| country*Environment | 220.803 | 5 | <.0001* |
| country*People | 74.731 | 5 | <.0001* |

# 3. DCE Gender interaction term model

**Choice Model: Main effect + Gender interaction**

**Effect Summary**

Supplementary Table 13 Choice model main effect and gender interaction, effect summary, parameter estimates and likelihood ratio tests

| **Source** | **Logworth** |  | **PValue** |
| --- | --- | --- | --- |
| S1. What is your gender?*Environment | 30,090 | 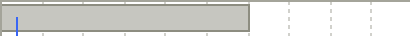 | 0,00000 |
| Face Mask | 22,734 | 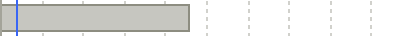 | 0,00000 |
| Environment | 19,399 | 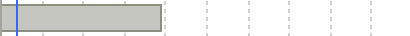 | 0,00000 |
| S1. What is your gender?*Face Mask | 6,437 | 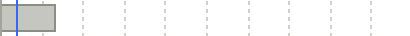 | 0,00000 |
| S1. What is your gender?*Distance | 2,594 | 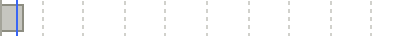 | 0,00254 |
| People | 2,349 | 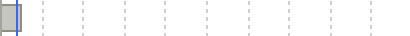 | 0,00448 |
| S1. What is your gender?*People | 1,661 | 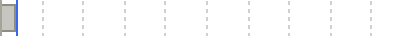 | 0,02182 |
| Distance | 1,408 | 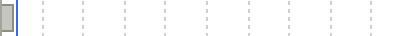 | 0,03908 |
| Duration | 0,697 | 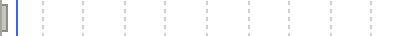 | 0,20088 |
| S1. What is your gender?*Duration | 0,319 | 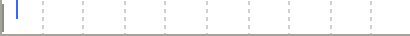 | 0,47997 |

**Parameter Estimates**

| **Term** | **Estimate** | **Std Error** | **Lower 95%** | **Upper 95%** |
| --- | --- | --- | --- | --- |
| Face Mask[Yes] | -0,354746831 | 0,0359631084 | -0,42512 | -0,286996 |
| Duration[15 minutes] | -0,045215301 | 0,0359546025 | -0,114753 | 0,0235101 |
| Distance[About 1 meter] | 0,073535483 | 0,0359546361 | 0,00481 | 0,1430731 |
| Environment[Outdoors, open air] | -0,336246911 | 0,0359590556 | -0,406351 | -0,268156 |
| People[2 people] | -0,101768508 | 0,0357623673 | -0,170873 | -0,033181 |
| S1. What is your gender?[Female]*Face Mask[Yes] | -0,079569995 | 0,0362368350 | -0,147885 | -0,008674 |
| S1. What is your gender?[Female]*Duration[15 minutes] | -0,005270125 | 0,0362262690 | -0,07454 | 0,0647982 |
| S1. What is your gender?[Female]*Distance[About 1 meter] | 0,031185063 | 0,0362263780 | -0,038883 | 0,100455 |
| S1. What is your gender?[Female]*Environment[Outdoors, open air] | -0,143215388 | 0,0362334295 | -0,211867 | -0,072584 |
| S1. What is your gender?[Female]*People[2 people] | -0,042436650 | 0,0360011286 | -0,1115 | 0,0271362 |
| S1. What is your gender?[Male]*Face Mask[Yes] | -0,023556089 | 0,0362074170 | -0,091811 | 0,0472839 |
| S1. What is your gender?[Male]*Duration[15 minutes] | 0,007533802 | 0,0361976920 | -0,061678 | 0,0775465 |
| S1. What is your gender?[Male]*Distance[About 1 meter] | -0,005128558 | 0,0361977497 | -0,075141 | 0,0640838 |
| S1. What is your gender?[Male]*Environment[Outdoors, open air] | -0,020875821 | 0,0362032279 | -0,089465 | 0,0496982 |
| S1. What is your gender?[Male]*People[2 people] | -0,015775794 | 0,0359927402 | -0,084822 | 0,0537806 |

| AICc | 59143,862 |
| --- | --- |
| BIC | 59276,892 |
| -2*LogLikelihood | 59113,853 |
| -2*Firth LogLikelihood | 58981,906 |

Converged in Gradient

Firth Bias-Adjusted Estimates

**Likelihood Ratio Tests**

| **Source** | **L-R ChiSquare** | **DF** | **Prob>ChiSq** |
| --- | --- | --- | --- |
| Face Mask | 99,621 | 1 | <,0001*** |
| Duration | 1,636 | 1 | 0,2009 |
| Distance | 4,257 | 1 | 0,0391*** |
| Environment | 84,425 | 1 | <,0001*** |
| People | 8,079 | 1 | 0,0045*** |
| S1. What is your gender?*Face Mask | 29,645 | 2 | <,0001*** |
| S1. What is your gender?*Duration | 1,468 | 2 | 0,4800 |
| S1. What is your gender?*Distance | 11,948 | 2 | 0,0025** |
| S1. What is your gender?*Environment | 138,571 | 2 | <,0001*** |
| S1. What is your gender?*People | 7,650 | 2 | 0,0218* |

# 4. DCE Age interaction

Supplementary Table 14 Choice model main effect and age group interaction, effect summary, parameter estimates and likelihood ratio tests

| **Source** | **FDR LogWorth** |  | **FDR PValue** |
| --- | --- | --- | --- |
| Environment | 309.048 |  | 0.00000 |
| Face Mask | 267.789 |  | 0.00000 |
| People | 30.884 |  | 0.00000 |
| S2. What is your age?*Environment | 22.427 |  | 0.00000 |
| Distance | 12.342 |  | 0.00000 |
| S2. What is your age?*Face Mask | 2.110 |  | 0.00777 |
| Duration | 1.980 |  | 0.01046 |
| S2. What is your age?*People | 0.984 |  | 0.10384 |
| S2. What is your age?*Duration | 0.777 |  | 0.16722 |
| S2. What is your age?*Distance | 0.284 |  | 0.51984 |

**Parameter Estimates**

| **Term** | **Estimate** | **Std Error** |
| --- | --- | --- |
| Face Mask[Yes] | -0.390786474 | 0.0104982614 |
| Duration[15 minutes] | -0.028415184 | 0.0104869432 |
| Distance[About 1 meter] | 0.078928033 | 0.0104871977 |
| Environment[Outdoors, open air] | -0.416085304 | 0.0105021561 |
| People[2 people] | -0.122360733 | 0.0101548105 |
| S2. What is your age?[18-24]*Face Mask[Yes] | 0.014986995 | 0.0156378409 |
| S2. What is your age?[18-24]*Duration[15 minutes] | -0.032032940 | 0.0155913956 |
| S2. What is your age?[18-24]*Distance[About 1 meter] | 0.005743320 | 0.0155923932 |
| S2. What is your age?[18-24]*Environment[Outdoors, open air] | 0.094244414 | 0.0156284273 |
| S2. What is your age?[18-24]*People[2 people] | -0.031627111 | 0.0151639686 |
| S2. What is your age?[25-34]*Face Mask[Yes] | -0.001368718 | 0.0134843136 |
| S2. What is your age?[25-34]*Duration[15 minutes] | -0.020413341 | 0.0134576005 |
| S2. What is your age?[25-34]*Distance[About 1 meter] | 0.001272593 | 0.0134581912 |
| S2. What is your age?[25-34]*Environment[Outdoors, open air] | 0.038995931 | 0.0134857234 |
| S2. What is your age?[25-34]*People[2 people] | -0.009846742 | 0.0130127523 |
| S2. What is your age?[35-44]*Face Mask[Yes] | -0.027847389 | 0.0133218543 |
| S2. What is your age?[35-44]*Duration[15 minutes] | -0.001438709 | 0.0132972341 |
| S2. What is your age?[35-44]*Distance[About 1 meter] | -0.001814527 | 0.0132977977 |
| S2. What is your age?[35-44]*Environment[Outdoors, open air] | -0.007559555 | 0.0133253931 |
| S2. What is your age?[35-44]*People[2 people] | -0.001504179 | 0.0128015842 |
| S2. What is your age?[45-54]*Face Mask[Yes] | -0.041447888 | 0.0138613105 |
| S2. What is your age?[45-54]*Duration[15 minutes] | -0.024480890 | 0.0138371669 |
| S2. What is your age?[45-54]*Distance[About 1 meter] | 0.014681968 | 0.0138377582 |
| S2. What is your age?[45-54]*Environment[Outdoors, open air] | -0.042773536 | 0.0138668183 |
| S2. What is your age?[45-54]*People[2 people] | 0.006359062 | 0.0132151261 |
| S2. What is your age?[55-64]*Face Mask[Yes] | 0.013672999 | 0.0176440433 |
| S2. What is your age?[55-64]*Duration[15 minutes] | 0.004761158 | 0.0176001318 |
| S2. What is your age?[55-64]*Distance[About 1 meter] | 0.029230562 | 0.0176027335 |
| S2. What is your age?[55-64]*Environment[Outdoors, open air] | -0.112602881 | 0.0176970021 |
| S2. What is your age?[55-64]*People[2 people] | -0.033107112 | 0.0167955037 |

| AICc | 59161.987 |
| --- | --- |
| BIC | 59428.03 |
| -2*LogLikelihood | 59101.952 |
| -2*Firth LogLikelihood | 58833.524 |

Converged in Gradient

Firth Bias-Adjusted Estimates

**Likelihood Ratio Tests**

| **Source** | **L-R ChiSquare** | **DF** | **Prob>ChiSq** |
| --- | --- | --- | --- |
| Face Mask | 1228.865 | 1 | <.0001* |
| Duration | 7.192 | 1 | 0.0073* |
| Distance | 53.752 | 1 | <.0001* |
| Environment | 1420.110 | 1 | <.0001* |
| People | 139.230 | 1 | <.0001* |
| S2. What is your age?*Face Mask | 16.916 | 5 | 0.0047* |
| S2. What is your age?*Duration | 8.106 | 5 | 0.1505 |
| S2. What is your age?*Distance | 4.208 | 5 | 0.5198 |
| S2. What is your age?*Environment | 116.795 | 5 | <.0001* |
| S2. What is your age?*People | 9.736 | 5 | 0.0831 |

# 5. DCE CRT Models

**Choice Model: Main effect + CRT Reflective interaction**

Supplementary Table 15 Choice model main effect and CRT Reflective score interaction, effect summary, parameter estimates and likelihood ratio tests

| **Source** | **FDR LogWorth** | **FDR PValue** |
| --- | --- | --- |
| Face Mask | 1455.005 | 0.00000 |
| Environment | 1096.745 | 0.00000 |
| CRT_Refl*Environment | 89.591 | 0.00000 |
| People | 41.131 | 0.00000 |
| CRT_Refl*Face Mask | 17.758 | 0.00000 |
| CRT_Refl*People | 16.689 | 0.00000 |
| Distance | 15.646 | 0.00000 |
| CRT_Refl*Distance | 8.908 | 0.00000 |
| Duration | 5.484 | 0.00000 |
| CRT_Refl*Duration | 2.248 | 0.00565 |

**Parameter Estimates**

| **Term** | **Estimate** | **Std Error** | **Lower 95%** | **Upper 95%** |
| --- | --- | --- | --- | --- |
| Face Mask[Yes] | -0.365242619 | 0.0070321549 | -0.37905 | -0.351485 |
| Duration[15 minutes] | -0.032750346 | 0.0070168444 | -0.046511 | -0.019006 |
| Distance[About 1 meter] | 0.057758187 | 0.0070171121 | 0.0440141 | 0.0715201 |
| Environment[Outdoors, open air] | -0.319058798 | 0.0070342523 | -0.332869 | -0.305296 |
| People[2 people] | -0.092017564 | 0.0067728706 | -0.105298 | -0.07875 |
| CRT_Refl*Face Mask[Yes] | -0.027759538 | 0.0031993737 | -0.034057 | -0.021515 |
| CRT_Refl*Duration[15 minutes] | -0.008765108 | 0.0031834833 | -0.015021 | -0.002541 |
| CRT_Refl*Distance[About 1 meter] | 0.019291338 | 0.0031840780 | 0.0130671 | 0.0255496 |
| CRT_Refl*Environment[Outdoors, open air] | -0.062289452 | 0.0032157847 | -0.068622 | -0.056015 |
| CRT_Refl*People[2 people] | -0.024493137 | 0.0028809659 | -0.030147 | -0.018854 |

| AICc | 58820.778 |
| --- | --- |
| BIC | 58909.467 |
| -2*LogLikelihood | 58800.774 |
| -2*Firth LogLikelihood | 58689.849 |

Converged in Gradient

Firth Bias-Adjusted Estimates

**Likelihood Ratio Tests**

| **Source** | **L-R ChiSquare** | **DF** | **Prob>ChiSq** |
| --- | --- | --- | --- |
| Face Mask | 2903.585 | 1 | <.0001* |
| Duration | 21.845 | 1 | <.0001* |
| Distance | 68.064 | 1 | <.0001* |
| Environment | 2186.745 | 1 | <.0001* |
| People | 185.564 | 1 | <.0001* |
| CRT_Refl*Face Mask | 78.326 | 1 | <.0001* |
| CRT_Refl*Duration | 7.660 | 1 | 0.0056* |
| CRT_Refl*Distance | 37.347 | 1 | <.0001* |
| CRT_Refl*Environment | 408.523 | 1 | <.0001* |
| CRT_Refl*People | 73.109 | 1 | <.0001* |

**Choice Model: Main effect + CRT Intuitive score interaction**

Supplementary Table 16 Choice model main effect and CRT-Intuitive score interaction, effect summary, parameter estimates and likelihood ratio tests

| **Source** | **FDR LogWorth** | **FDR PValue** |
| --- | --- | --- |
| Environment | 859.845 | 0.00000 |
| Face Mask | 251.371 | 0.00000 |
| People | 38.687 | 0.00000 |
| Distance | 11.536 | 0.00000 |
| CRT_Int*Face Mask | 4.434 | 0.00004 |
| Duration | 3.003 | 0.00099 |
| CRT_Int*Environment | 1.390 | 0.04071 |
| CRT_Int*People | 0.346 | 0.45119 |
| CRT_Int*Distance | 0.309 | 0.49034 |
| CRT_Int*Duration | 0.218 | 0.60537 |

**Parameter Estimates**

| **Term** | **Estimate** | **Std Error** | **Lower 95%** | **Upper 95%** |
| --- | --- | --- | --- | --- |
| Face Mask[Yes] | -0.362166502 | 0.0110196692 | -0.383815 | -0.34062 |
| Duration[15 minutes] | -0.037646479 | 0.0109856219 | -0.059197 | -0.016135 |
| Distance[About 1 meter] | 0.077846065 | 0.0109865982 | 0.056335 | 0.0994002 |
| Environment[Outdoors, open air] | -0.437824130 | 0.0110299024 | -0.459496 | -0.416262 |
| People[2 people] | -0.138761068 | 0.0105384715 | -0.159431 | -0.118124 |
| CRT_Int*Face Mask[Yes] | -0.014083005 | 0.0032886586 | -0.020529 | -0.007638 |
| CRT_Int*Duration[15 minutes] | -0.001694044 | 0.0032781140 | -0.008119 | 0.0047304 |
| CRT_Int*Distance[About 1 meter] | 0.002524234 | 0.0032784037 | -0.003901 | 0.0089497 |
| CRT_Int*Environment[Outdoors, open air] | 0.007203302 | 0.0032897872 | 0.0007561 | 0.0136512 |
| CRT_Int*People[2 people] | 0.002863073 | 0.0031349412 | -0.003281 | 0.0090073 |

| AICc | 59251.533 |
| --- | --- |
| BIC | 59340.221 |
| -2*LogLikelihood | 59231.529 |
| -2*Firth LogLikelihood | 59121.222 |

Converged in Gradient

Firth Bias-Adjusted Estimates

**Likelihood Ratio Tests**

| **Source** | **L-R ChiSquare** | **DF** | **Prob>ChiSq** |
| --- | --- | --- | --- |
| Face Mask | 1153.322 | 1 | <.0001* |
| Duration | 11.789 | 1 | 0.0006* |
| Distance | 50.546 | 1 | <.0001* |
| Environment | 1713.791 | 1 | <.0001* |
| People | 174.942 | 1 | <.0001* |
| CRT_Int*Face Mask | 18.348 | 1 | <.0001* |
| CRT_Int*Duration | 0.267 | 1 | 0.6054 |
| CRT_Int*Distance | 0.593 | 1 | 0.4413 |
| CRT_Int*Environment | 4.798 | 1 | 0.0285* |
| CRT_Int*People | 0.835 | 1 | 0.3609 |

# 6. DCE Interaction with perceived fright of COVID19 (PFC)

**Perceived Fright of Covid19 was measured with the following item:**

How did you perceive the Coronavirus\Covid situation? To me, the Coronavirus\Covid is ....

not frightening (1) 2 3 4 frightening(5)

Supplementary Table 17 Choice model main effect and Perceived Fright of Covid19 score (PFC) interaction, effect summary, parameter estimates and likelihood ratio tests

| **Source** | **L-R ChiSquare** | **DF** | **Prob>ChiSq** |
| --- | --- | --- | --- |
| Face Mask | 5337,757 | 1 | <,0001*** |
| Duration | 61,907 | 1 | <,0001*** |
| Distance | 239,692 | 1 | <,0001*** |
| Environment | 6540,253 | 1 | <,0001*** |
| People | 590,087 | 1 | <,0001*** |
| M8C. COVID-19 threat perceptions and experiences_not frightening  - frightening*Face Mask | 659,221 | 4 | <,0001*** |
| M8C. COVID-19 threat perceptions and experiences_not frightening  - frightening*Duration | 1,286 | 4 | 0,8637 |
| M8C. COVID-19 threat perceptions and experiences_not frightening  - frightening*Distance | 9,063 | 4 | 0,0595 |
| M8C. COVID-19 threat perceptions and experiences_not frightening  - frightening*Environment | 65,444 | 4 | <,0001*** |
| M8C. COVID-19 threat perceptions and experiences_not frightening  - frightening*People | 24,915 | 4 | <,0001*** |

| **Term** | **Estimate** | **Odds ratios** | **Probabilities** |
| --- | --- | --- | --- |
| Face Mask[Yes] | -0.388472268 | 0.68 | -32% |
| Duration[15 minutes] | -0.043845065 | 0.96 | -4% |
| Distance[About 1 meter] | 0.086074210 | 1.09 | +9% |
| Environment[Outdoors, open air] | -0.424140363 | 0.65 | -35% |
| People[2 people] | -0.128485299 | 0.88 | -12% |
| frightening[1]*Face Mask[Yes] | 0.257822643 | 1.29 | +29% |
| frightening[1]*Duration[15 minutes] | 0.011255443 | n.s. | n.s |
| frightening[1]*Distance[About 1 meter] | -0.027431958 | n.s | n.s |
| frightening[1]*Environment[Outdoors, open air] | 0.079891566 | 1.08 | +8% |
| frightening[1]*People[2 people] | 0.047102835 | 1.05 | +5% |
| frightening[2]*Face Mask[Yes] | 0.047992426 | 1.05 | +5% |
| frightening[2]*Duration[15 minutes] | -0.003380172 | n.s. | n.s |
| frightening[2]*Distance[About 1 meter] | 0.007756249 | n.s. | n.s |
| frightening[2]*Environment[Outdoors, open air] | -0.028773125 | 0.97 | -3% |
| frightening[2]*People[2 people] | 0.004184483 | 1.004 | = |
| frightening[3]*Face Mask[Yes] | -0.020921351 | 0.97 | -3% |
| frightening[3]*Duration[15 minutes] | -0.005131870 | n.s. | n.s |
| frightening[3]*Distance[About 1 meter] | -0.002861587 | n.s. | n.s |
| frightening[3]*Environment[Outdoors, open air] | 0.022387426 | 1.02 | +2% |
| frightening[3]*People[2 people] | -0.001930773 | 0.99 | -1% |
| frightening[4]*Face Mask[Yes] | -0.123928451 | -0.88 | -12% |
| frightening[4]*Duration[15 minutes] | -0.004786402 | n.s. | n.s |
| frightening[4]*Distance[About 1 meter] | 0.022852163 | n.s. | n.s |
| frightening[4]*Environment[Outdoors, open air] | -0.039003388 | 0.96 | -4% |
| frightening[4]*People[2 people] | -0.032923260 | 0.97 | -3% |
